# Bone marrow mesenchymal stromal cell-derived extracellular matrix displays altered glycosaminoglycan structure and impaired functionality in Myelodysplastic Syndromes

**DOI:** 10.3389/fonc.2022.961473

**Published:** 2022-09-08

**Authors:** Amanpreet Kaur Bains, Lena Behrens Wu, Jennifer Rivière, Sandra Rother, Valentina Magno, Jens Friedrichs, Carsten Werner, Martin Bornhäuser, Katharina S. Götze, Michael Cross, Uwe Platzbecker, Manja Wobus

**Affiliations:** ^1^ Medical Department I, Haematology and Cell Therapy, University of Leipzig Medical Center, Leipzig, Germany; ^2^ Department of Medicine I, University Hospital Carl Gustav Carus, Technische Universität, Dresden, Germany; ^3^ Department of Medicine III, Hematology/Oncology, School of Medicine, Klinikum rechts der Isar, München, Technical University of Munich, Munich, Germany; ^4^ Center for Molecular Signaling Präklinisches Zentrum für Molekulare Signalverarbeitung (PZMS), Saarland University School of Medicine, Homburg, Germany; ^5^ Max Bergmann Center of Biomaterials, Leibniz Institute of Polymer Research Dresden, Technische Universität (TU) Dresden, Dresden, Germany

**Keywords:** mesenchymal stromal cells, hematopoiesis, bone marrow, extracellular matrix, myelodysplastic syndromes

## Abstract

Myelodysplastic syndromes (MDS) comprise a heterogeneous group of hematologic malignancies characterized by clonal hematopoiesis, one or more cytopenias such as anemia, neutropenia, or thrombocytopenia, abnormal cellular maturation, and a high risk of progression to acute myeloid leukemia. The bone marrow microenvironment (BMME) in general and mesenchymal stromal cells (MSCs) in particular contribute to both the initiation and progression of MDS. However, little is known about the role of MSC-derived extracellular matrix (ECM) in this context. Therefore, we performed a comparative analysis of *in vitro* deposited MSC-derived ECM of different MDS subtypes and healthy controls. Atomic force microscopy analyses demonstrated that MDS ECM was significantly thicker and more compliant than those from healthy MSCs. Scanning electron microscopy showed a dense meshwork of fibrillar bundles connected by numerous smaller structures that span the distance between fibers in MDS ECM. Glycosaminoglycan (GAG) structures were detectable at high abundance in MDS ECM as white, sponge-like arrays on top of the fibrillar network. Quantification by Blyscan assay confirmed these observations, with higher concentrations of sulfated GAGs in MDS ECM. Fluorescent lectin staining with wheat germ agglutinin and peanut agglutinin demonstrated increased deposition of *N*-acetyl-glucosamine GAGs (hyaluronan (HA) and heparan sulfate) in low risk (LR) MDS ECM. Differential expression of *N*-acetyl-galactosamine GAGs (chondroitin sulfate, dermatan sulfate) was observed between LR- and high risk (HR)-MDS. Moreover, increased amounts of HA in the matrix of MSCs from LR-MDS patients were found to correlate with enhanced HA synthase 1 mRNA expression in these cells. Stimulation of mononuclear cells from healthy donors with low molecular weight HA resulted in an increased expression of various pro-inflammatory cytokines suggesting a contribution of the ECM to the inflammatory BMME typical of LR-MDS. CD34^+^ hematopoietic stem and progenitor cells (HSPCs) displayed an impaired differentiation potential after cultivation on MDS ECM and modified morphology accompanied by decreased integrin expression which mediate cell-matrix interaction. In summary, we provide evidence for structural alterations of the MSC-derived ECM in both LR- and HR-MDS. GAGs may play an important role in this remodeling processes during the malignant transformation which leads to the observed disturbance in the support of normal hematopoiesis.

## Introduction

Myelodysplastic syndromes (MDS) comprise a heterogeneous group of clonal hematological disorders characterized by the presence of peripheral cytopenias and an increased risk of transformation into acute myeloid leukemia (AML). The World Health Organisation’s International Prognostic Scoring System (IPSS) classifies MDS into low-, intermediate- and high-risk groups based on the number of dysplastic lineages, cytogenetic abnormalities, presence or absence of ring sideroblasts and the percentage of blasts in the bone marrow (BM) and peripheral blood (1). In some MDS categories, a specific genotype e.g. cytogenetic aberration and mutation in the hematopoietic stem and progenitor cells (HSPCs) correlates at least partially with the phenotype and prognosis of the disease (1). MDS are progressive diseases that evolve through a multi-step pathogenesis. Early stages of the disease (low- to intermediate-risk) harbor rare, multipotent stem cells with somatic genomic mutations (2). Disease progression towards AML is a result of continuation of this process involving hypermethylation, silencing of tumor suppressor genes and activation of oncogenes.

The involvement of the bone marrow microenvironment (BMME) in disease initiation, progression and treatment response has become increasingly apparent. A range of molecules and signaling mechanisms have been shown to be altered in the mesenchymal stromal cells (MSCs) that are major components of the BMME (3, 4). Together with matrix-bound morphogens MSCs demonstrated involvement in the regulation of HSPCs homeostasis, mobilisation, and homing (5). The profound immune dysregulation and sustained inflammation mediated by myeloid-derived suppressor cells and through Toll-like receptor signaling are further factors that contribute to ineffective hematopoiesis and drive disease progression (6, 7).

Another fundamental component of the BMME is the extracellular matrix (ECM), a complex and hydrated meshwork of proteins and carbohydrates which forms a spatiotemporally adaptive, three-dimensional architectural scaffold that defines tissue integrity and tissue/organ boundaries across scales, as well as cell polarity. The ECM is composed mainly of collagens, glycoproteins and glycosaminoglycans/proteoglycans that are secreted by the cells into the immediate BMME (8). Here, matrix components modulate HSPC behaviour by structural organisation of hematopoietic-stromal cell interactions and paracrine signalling, biomechanical cues as well as by the sequestration and presentation of matrix-bound cytokines (9, 10). In this way, the ECM contributes to the establishment of niches in which proliferation and differentiation are balanced to maintain both the constant provision of mature blood cells and the maintenance of a pool of undifferentiated HSPCs throughout life.

To date, our understanding of the ECM in hematological malignancies is mainly derived from research of the role of collagens and certain glycoproteins (11–13). In contrast, relatively little is known about the role of glycosaminoglycans (GAGs). GAGs are negatively charged linear polysaccharides containing alternating repeats of uronic acid and hexose sugar. Chondroitin sulfate (CS) and hyaluronan (HA) are the major GAGs present in the BM (14, 15). Except HA, all GAGs are covalently linked to a core scaffold protein, but the GAG chains mainly determine the function of the proteoglycan. GAGs are an integral part of the ECM, and by virtue of their hydrophilic nature and affinity for certain proteins they are instrumental in maintaining hydration and morphogen gradients. GAGs are also potent stimulators and regulators of different signalling pathways, including WNT, phosphoinositide 3-kinase (PI3K)/protein kinase B (Akt) and Mitogen-Activated Protein Kinases (MAPK), Receptor Activator of NF-κB Ligand/osteoprotegerin, SMAD, and various inflammatory pathways (16–18).

Although the influence of the BMME on malignant transformation has been investigated in some detail, the role of ECM, and especially of GAGs, has received relatively little attention to date. The characterisation of ECM biology in MDS will identify properties and features relevant to a better understanding of the disease and the improvement of therapeutic strategies.

In this study, we demonstrate disease stage-specific compositional and structural differences between the ECM of MDS and healthy MSCs in terms of GAG composition, leading to a modulation of integrin expression and cytoskeletal arrangement in co-cultured normal HSPCs. Thus, we provide novel data concerning the role of GAGs and ECM remodelling during malignant transformation in the BM.

## Material and methods

### Patient cohort

MSCs of MDS patients (n= 10, IPSS-R lower (LR) including intermediate risk 1, and higher (HR) risk including intermediate risk 2) with normal karyotype were isolated from BM aspirates after obtaining informed written consent. The control cohort (n=6) was collected from hematologically healthy individuals who were undergoing hip replacement surgery. These samples were screened for the absence of clonal hematopoiesis (CHIP) (19). The median age was 67± 5.4 years for the LR MDS group, 62± 2.8 years for the HR MDS group and 65± 2.8 years for the healthy donors (**Supplementary Table S1**).

### MSC culture and ECM generation

MSCs were isolated from BM aspirates as described previously (20, 21). The cells were expanded in Dulbecco’s modified Eagle’s medium-low glucose (DMEM, Gibco, Germany) with 10% fetal bovine serum (FBS, Gibco, Germany) and characterized according to the criteria of the International Society for Cellular Therapy (22). MSCs were used in the second to fourth passage for all experiments. To yield cell-free ECM substrates, MSCs were seeded on poly-octadecene-alt-maleic anhydride (POMA) and fibronectin coated glass slides as described previously (23). Decellularization was performed at day 10, unless otherwise noted, by using an aqueous solution of NH_4_OH (Merck, Germany), and DNA residues were removed by incubating the samples with DNase I (Merck, Germany). Samples were finally washed in PBS and stored in Hank’s Balanced Salt Solution (HBSS) with calcium and magnesium (Merck, Germany) at 4°C until further use for no more than a month.

### Isolation of CD34^+^ HSPCs

CD34^+^ HSPCs were isolated from leukapheresis of healthy donors after obtaining informed written consent. The cells were purified by immunomagnetic sorting using CD34 MicroBead Kit UltraPure (Miltenyi Biotec, Germany). The viability was determined by live-dead-discrimination using Countess II Automated Cell Counter (Thermo Fisher, Germany) and the purity of at least 98% was confirmed by flow cytometry.

### Hematopoietic cell culture on ECM substrates

CD34^+^ HSPCs were added in CellGro medium (CellGenix, Germany) containing 2.5% FCS as well as SCF, FLT3-L and IL-3 (2.5 ng/ml each, all from Miltenyi Biotec, Germany). After seven days, cells were either collected for further analysis of clonogenicity or they were fixed on the matrices by 4% PFA for immunofluorescence staining.

### Clonogenic assays

Colony forming unit (CFU) assays were carried out using CD34^+^ HSPCs harvested after seven days of culture on different matrix samples, with 500 cells being plated in Stem MACS HSC-CFU complete with Epo (Miltenyi Biotec). Colonies were counted after 2 weeks and classified with the StemVision system (Stem Cell Technologies, Germany). Each sample was run in duplicates.

### Atomic force microscopy

AFM measurements were performed using a Nanowizard I AFM (JPK Instruments) mounted on an inverted optical microscope (Observer A1, Zeiss) as described previously (24). For elasticity and height measurements, a tipless, 200 µm long, V-shaped cantilever with a nominal spring constant of 0.06 N/m (NPN-TR-TL-Au, Bruker) was used. The cantilever was calibrated using the equipartition theorem (Quelle 50).

The Young’s modulus was extracted from approach force-distance curves using AFM data processing software (JPK Instruments). To measure the thickness of hydrated ECM substrates a defined scratch was applied to the substrate to analyze the difference in height over the scratch area. To create a scratch, ECM substrates were first dehydrated under a laminar flow of nitrogen after three washes in double distilled water. Using a scalpel, a straight scratch was drawn into the decellularized ECM. The substrate was rehydrated in PBS and processed for AFM measurement. The height of the matrices was determined by performing a 100 µm x 20 µm field scan perpendicular to the scratch at 1 µm/s scan velocity.

### Scanning electron microscopy

To prepare samples for SEM, decellularized ECM on glass coverslips were washed with PBS and fixed in 0.1 M cacodylate buffer, 2% paraformaldehyde, 2% glutaraldehyde, 0.2% ruthenium red, rinsed in 0.1 M cacodylate buffer containing 7.5% sucrose and 0.1% ruthenium red, followed by post-fixation in 0.1 M cacodylate buffer containing 1% osmium tetroxide and 0.05% ruthenium red. After washing in double distilled water and critical point drying (Bal-Tec CPD 030, Bal-Tec), samples were sputtered with 3 nm platinum (Bal-Tec SCD 500, Bal-Tec) and examined using a scanning electron microscope (Zeiss Ultra Plus, Carl Zeiss Microscopy, Germany).

### Quantification of sulfated GAGs by Blyscan assay

Sulfated Glycosaminoglycan (sGAG) of the ECM substrates were measured using the Blyscan assay kit (Biocolor, UK). Briefly, sGAGs were extracted using papain extraction reagent (0.2 M sodium phosphate buffer pH 6.4, containing 0.1 M sodium acetate, 0.01 M Na_2_EDTA, 0.00 5 M cysteine HCL and 0.1mg/ml papain (all from Sigma-Aldrich, Germany) at 65°C for 3 hours. Blyscan reagent (1,9-dimethyl-methylene blue) was added to an aliquot (100 µl) of the sample and was incubated at room temperature for 30 minutes to allow the formation of the dye-complex. The complex was pelleted down, and the dye was eluted by adding a sodium salt of anionic surfactant. The eluted dye was measured spectrophotometrically at 660 nm in duplicates using Biotek 800™ TS Absorbance Reader. Total sGAG content was later calculated using a calibration curve of known concentrations of sGAGs supplied by the manufacturer.

### Lectin and immunostaining of ECM and HSPCs

Lectin PNA Alexa Fluor™ 488 Conjugate (Invitrogen; 1:20) and Lectin WGA Alexa Fluor™ 594 Conjugate were used to stain *N*-acetyl-galactosamine GAGs and *N*-acetyl-glucosamine GAGs, respectively. A mouse anti-human CS antibody (CS56, Sigma-Aldrich, Germany) and goat anti-mouse Alexa Fluor™ 488 (Thermo-Fisher, Germany) secondary antibody were used to detect CS deposition. Briefly, the ECM substrates were fixed in 4% PFA for 15 minutes and blocked using 2% bovine serum albumin (BSA)/PBS (Merck, Germany) for 1 hour at room temperature. The substrates were incubated with the aforementioned lectins or primary and secondary antibodies. The stained substrates were mounted using fluorescence mounting medium and stored in dark until imaging. The substrates were imaged using a Keyence BZ-X810 microscope and fluorescence intensity was measured using Image J (NIH).

To verify the specificity of the lectin and CS antibody staining, ECM substrates were subjected to pretreatment with the GAG degrading enzymes chondroitinase ABC from *Proteus vulgaris* (Sigma) and heparinase-I from *Flavobacterium heparinum* (Sigma). Briefly, the samples were incubated with chondroitinase ABC (2 units/ml) and heparinase-I (50 units/ml) in 50 mM Tris, 0.02% BSA, 60 mM sodium acetate buffer for 24 hours at 37°C and pH 8.0 for digestion of chondroitin sulfate, dermatan sulfate and heparan sulfate. After 24 hours, the ECM substrates were further subjected to chondroitinase ABC (2 units/ml) for 48 hours at 37°C and pH 6.8 for digestion of hyaluronan. ECM samples in the buffer solution without GAG degrading enzymes were used as controls.

HSPCs on ECM preparations were fixed with 4% PFA for F-actin and integrin staining. Cells were permeabilized using PBS containing 0.1% Triton X-100 (T-PBS), blocked with T-PBS containing 10% FCS and 1% human serum albumin (IF-Buffer) and incubated over night at 4°C with Alexa Fluor 488 phalloidin for actin staining and monoclonal mouse anti-human-ITGαVβ3 (Abcam, Germany), followed by polyclonal sheep-anti-rabbit-Cy3 (Sigma-Aldrich, Germany). After counterstaining with DAPI and mounting, the cell evaluation was performed by confocal microscopy (LSM880 Airy, Carl Zeiss Microscopy, Germany). ITGαVβ3 fluorescence intensity was measured using Image J.

### Quantification of HA in the ECM substrates

Cells were cultivated on POMA/fibronectin-coated glass cover slides in 6-well plates for three weeks prior to removal of the cells with 20 mM ammonia and DNaseI treatment. The dried matrices were enzymatically degraded by overnight incubation with papain (1 mg/ml) in PBS at 60°C. The amounts of hyaluronan in the cell-derived matrices were quantified by sandwich enzyme-linked immunosorbent assay (ELISA) following the manufacturer´s instructions (R&D Systems). In brief, 96-well ELISA plates were overnight incubated with recombinant human aggrecan (500 ng/ml) dissolved in PBS. Afterwards, the washed plates were incubated with the sample solutions for 120 minutes (100 µl per well). Bound hyaluronan was quantified using biotinylated recombinant human aggrecan (400 ng/ml, R&D Systems, Germany) diluted in 1% BSA in PBS. The calibration curves for comparison ranged from 0-90 ng/ml hyaluronan diluted with 1% BSA in PBS.

### Separation and visualization of ECM GAGs

The cell-derived ECM GAGs were extracted as described previously (25). After papain treatment as stated in the section above, 100% (w/v) trichloroacetic acid was added to the sample solutions to reach a final concentration of 6% for protein removal. Precipitated peptides and proteins were removed after 120 minutes of incubation on ice and centrifugation for 30 minutes at 4°C (15000 g). GAGs were extracted by adding 4 vol of 98% ethanol. After overnight precipitation, the samples were centrifugated at 4°C for 30 minutes (15000 g) and the supernatants were discarded. The dried pellets were dissolved in water and loaded onto agarose gels (1%, 0.5 cm thickness) casted in 40 mM Tris-acetate, 1 mM EDTA buffer (TAE buffer). 5 µl hyaluronan molecular weight ladders (amsbio) and 3-6 µg of commercially available GAGs served as controls. Low- and high-molecular-weight hyaluronan (LMW-HA, HMW-HA) and CS-A/C mixture were obtained from Innovent e.V. (Jena), while heparin (porcine), CS-A (bovine) and CS-C (shark) were purchased from Sigma-Aldrich. Prior to the sample run, the agarose gel was pre-run for 6 hours at 80 V in TAE buffer. The gel run separating the ECM GAGs was performed at 100 V for 3 hours. Afterwards, the GAGs were stained overnight with Stains-all (5 mg in 100 ml 50% ethanol) in the dark. The gel was washed with water before destaining by exposing to light until the background staining is reduced. The gel was imaged using a scanner.

### HA stimulation of mononuclear cells

Healthy bone marrow mononuclear cells (5x10^6^ cells/ml) were stimulated with 10 µg/ml of low molecular weight HA (10-20 kDa) (Lifecore Biomedical, USA) for 24 hours in CellGro medium containing 2.5% FCS as well as SCF, FLT3-L and IL-3 (2.5 ng/ml each). Unstimulated cells were used as a control. mRNA was isolated using RNeasy micro kit (Qiagen). The mRNA expression of different inflammatory genes relevant to MDS such as IL6, IL1ß, IL18, NLRP3, PYCARD and S100A9 was analyzed by quantitative real-time PCR.

### Real-time PCR

Total RNA was isolated from MSCs after five days of culture before seeding them on POMA slides for ECM generation as well as from 24h stimulated MNCs using RNeasy Mini Kit (Qiagen, Germany) and reverse transcribed into cDNA using RevertAid cDNA synthesis kit (Thermo-Fisher, Germany) with oligo-dT primers. Relative target quantity was determined using the comparative CT(ΔΔCT) method. RT-PCR was performed using SYBR Green/ROX PCR master mix (Thermo-Fisher, Germany) and target specific primers (**Supplementary Table S2**) on a 7500 Real-time PCR cycler (Applied Biosystems, Germany). Amplicons were normalized to U6, respectively. All samples were run in duplicates.

### Statistical analysis

Statistical analysis was performed using GraphPad Prism software version 7./8.01. (GraphPad Software). Data are presented as mean ± standard deviation (SD). All experiments were repeated between three and five times. For relative quantification measurements, one sample t-test and unpaired t-test were performed. For multiple group comparisons, ANOVA followed by Tukey’s posthoc test was performed. A p-value of less than 0.05 was regarded as being statistically significant.

## Results

### MDS MSC ECM displays a more compact ultrastructure

**Figure 1:**
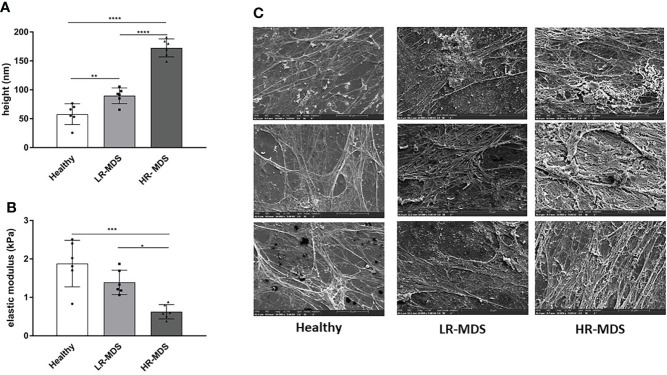
Ultrastructural ECM analyses. **(A)** Height and **(B)** elastic modulus measurement of ECM from healthy donor, LR-MDS and HR-MDS MSC. Bars represent mean ± SD in triplicate for N=3 samples per group. *p< 0.05, **p< 0.01, ***p< 0.001, ****p< 0.0001, analyzed by one-way ANOVA. **(C)** Representative electron micrographs for each sample group, 10.000x.

An accurate evaluation of the topographical and mechanical properties of the MSC-derived ECM was performed using AFM-imaging and –nanoindentation, respectively. The thickness was found to be significantly increased in MDS MSC-derived ECM, in particular in HR-MDS. Whereas healthy MSC-derived matrices displayed a height of 57.8 ± 16.54 nm, LR-MDS ECM were 89.70 ± 12.44 nm and HR-MDS ECM 172.5 ± 14.2 nm (**Figure 1A**). The elasticity as determined by the Young’s modulus was lower in LR-MDS than in healthy MSC ECM (1.39 ± 0.29 vs. 1.88 ± 0.55 kPa) and reached a significantly lower value in HR-MDS (0.62 ± 0.17 kPa) suggesting that MSCs secreted a more compliant matrix under malignant conditions (**Figure 1B**).

Electron micrographs of the decellularized matrices demonstrated that both LR- and HR-MDS MSCs deposit a denser ECM network with thick fibril bundles covered by a layer of tightly packed micro-fibrils. Moreover, GAG structures were detectable in high frequency in MDS ECM as white sponge-like arrangements on top of the fibrillar network (**Figure 1C**).

### MDS MSC-derived ECM have an altered GAG profile

**Figure 2:**
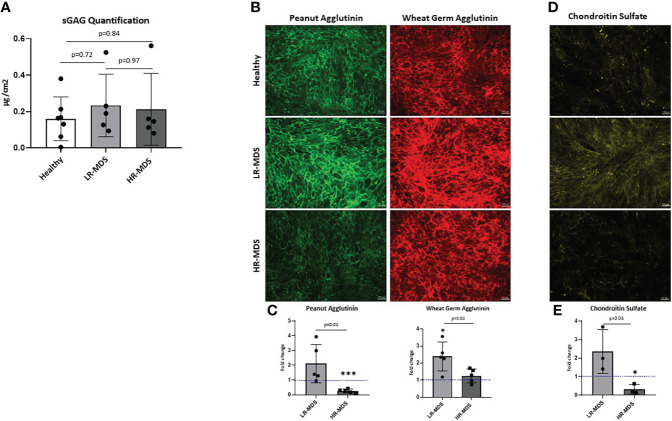
Analysis of *N*-acetyl-glucosamine and *N*-acetyl-galactosamine GAGs as well as of CS in ECM. **(A)** Quantification of sulfated GAGs of ECM from healthy donor, LR-MDS and HR-MDS MSC using Blyscan assay. **(B)** Representative images from the lectin staining using Wheat germ agglutinin (green) which binds to *N*-acetyl-glucosamine GAGs (HA, heparan sulfate and keratan sulfate) and sialic acid and peanut agglutinin (red) binds to *N*-acetyl-galactosamine and terminal ß-galactose containing GAGs (CS, dermatan sulfate and keratan sulfate) from each sample group. **(C)** Semi-quantitative analysis of relative fluorescence intensity in the immunofluorescence images by ImageJ analysis. Bars represent mean ± SD of N=5 MDS samples, each measured in an independent experiment and expressed as fold change relative to the accompanying healthy MSC ECM control, shown by the dotted line with the ordinate value of 1. Significant differences to the control value as determined by one sample t-test are indicated by an asterisk above the bar: *p< 0.05, ***p< 0.001. and differences between the two MDS groups as determined by unpaired t-test are shown by the p value above the respective bars. **(D)** Representative images from chondroitin sulfate of ECM from healthy donor, LR-MDS and HR-MDS MSC. **(E)** Semi-quantitative analysis of relative fluorescence intensity in the immunofluorescence images by ImageJ analysis. Bars represent mean ± SD of N=3 MDS samples measured in independent experiments and expressed as fold change relative to the accompanying healthy MSC ECM control shown by the dotted line at ordinate value 1. Significant differences to the control value as determined by one sample t-test are indicated by an asterisk above the bar: * = p< 0.05 and differences between the two MDS groups as determined by unpaired t-test are shown by the p value above the respective bars.

The total sulfated GAG content in the generated ECM was quantified by Blyscan assay. Although there was a tendency for increased GAG content in MDS MSC-derived ECM, the differences were not statistically significant (**Figure 2A**). Different groups of GAGs, e.g. *N*-acetyl-glucosamine and *N*-acetyl-galactosamine containing GAGs were analyzed by immunofluorescence staining of the different matrices using lectins conjugated to fluorophores. PNA staining demonstrated higher deposition of *N*-acetyl-galactosamine GAGs (chondroitin sulfate (CS), dermatan sulfate) in LR-MDS ECM compared to healthy samples, but also compared to HR-MDS ECM which displayed only weak staining (**Figures 2B, C**). WGA staining demonstrated significantly increased deposition of *N*-acetyl-glucosamine GAGs (hyaluronic acid (HA) and heparan sulfate) in LR-MDS ECM and a slight elevation in HR-MDS ECM compared to healthy controls (**Figures 2B, C**). Specificity of the lectin staining was confirmed by pretreatment of the ECM substrates with GAG degrading enzymes (**Supplementary Figures S1A, B**).

Next, the changes observed by PNA staining were further analysed for specific deposition of CS using a CS recognizing antibody (CS56). In line with the obtained results, we detected strongest CS staining in LR-MDS matrices, whereas ECM derived from HR-MDS contained only marginal amounts which were even lower than in healthy samples (**Figures 2D, E**). Chondroitinase ABC pretreatment of the ECM samples confirmed the CS-specific immunostaining (**Supplementary Figure S1C**).

**Figure 3:**
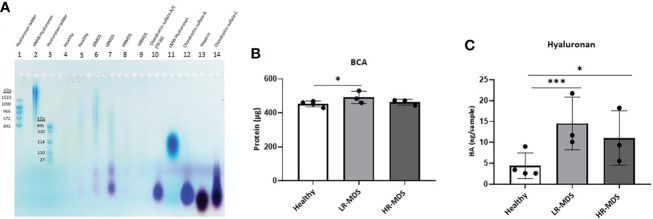
HA analysis in ECM samples. **(A)** Representative images of the stains all gel staining to characterize different GAGs in the ECM from each sample group. **(B)** Quantification of total protein of the ECM from healthy donor, LR-MDS and HR-MDS MSC using BCA assay. **(C)** Total HA quantification of the ECM from each sample group using ELISA. Bars represent mean ± SD in triplicate for N=4 for healthy donor MSC derived ECM and N=3 for per MDS sample group. *p< 0.05, ***p< 0.001 analyzed by one-way ANOVA.

In addition, separation of extracted GAGs by agarose gel electrophoresis showed the presence of CS-A in healthy, LR-MDS and HR-MDS samples, with the most intense bands corresponding to LR-MDS ECMs (**Figure 3A**).

### Increased levels of HA are present in MDS ECM

The changes observed by WGA staining were further analysed for HA deposition in the ECM using electrophoresis and stains all dye staining as well as ELISA. HA analysis by agarose gel electrophoresis showed the strongest HA bands in LR-MDS samples and fainter bands in healthy and HR-MDS ECMs (**Figure 3A**). Compared to HA molecular weight ladders, a wide distribution in HA molecular weight was observed for all samples reaching from > 1510 kDa to about 110 kDa. However, variations between donors were apparent, especially between LR-MDS patient samples, where LMW-HA with a molecular weight of below 110 kDa was detectable in one of the donors.

Protein quantification by BCA showed increased amounts in LR-MDS ECM compared to healthy and HR-MDS samples (**Figure 3B**). The HA ELISA demonstrated a significant increase in the total HA content in the LR-MDS and HR-MDS ECM compared to the healthy donor ECM. Although not significant, a tendency towards lower HA deposition was observed in HR-MDS ECM when compared to LR-MDS (**Figure 3C**).

### MDS MSCs show altered expression of GAG modifying enzymes

**Figure 4:**
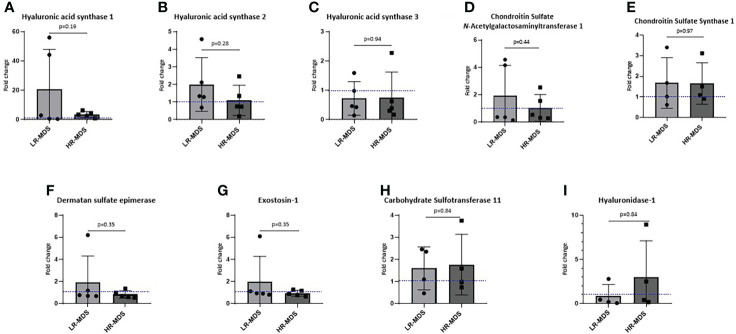
Expression analysis of GAG modifying enzymes in MSCs. Gene expression of HAS1 **(A)**, HAS2 **(B)**, HAS3 **(C)**, CSGALNacT1 **(D)**, chondroitin sulfate synthase-1 **(E)**, dermatan sulfate epimerase **(F)**, exostosin-1 **(G)**, carbohydrate sulfotransferase-11 **(H)** and hyaluronidase-1 **(I)** analyzed using quantitative real-time PCR. Bars represent mean ± SD for N=5 MDS samples expressed as fold change relative to the healthy MSC sample, shown as the dotted line at ordinate value of 1. Differences between the two MDS groups as determined by unpaired t-test are shown by the p value.

To investigate whether an increased deposition of HA and CS by MDS MSCs is associated with altered expression levels of GAG modifying enzymes, such as hyaluronan synthases (HAS1, HAS2, HAS3) and chondroitin sulfate *N*-acetylgalactosaminyltransferase 1 (CSGALNACT1), chondroitin sulfate synthase 1, carbohydrate sulfotransferase 11, dermatan sulfate epimerase, exostosin-1 quantitative real-time PCR was performed on MSCs before ECM generation. Interestingly, we observed not only different expression levels of HAS between MSCs from healthy donors and MDS MSCs, but also between the different MDS risk subtpyes, although the differences were not significant. Specifically, HAS1 expression was strongly increased in LR-MDS and remained above control levels in HR-MDS (**Figure 4A**), while HAS2 was up-regulated specifically in LR-MDS, being comparatively low in both healthy donor and HR-MDS MSCs (**Figure 4B**). HAS3 levels were specifically lower in HR-MDS when compared to LR-MDS and healthy controls (**Figure 4C**). However, there was no change in the enzymes involved in CS, dermatan sulfate and heparan sulfate biosynthesis and degradation pathways (**Figures 4D-I**).

### Low Molecular weight HA stimulates inflammatory pathways in MNC

**Figure 5:**
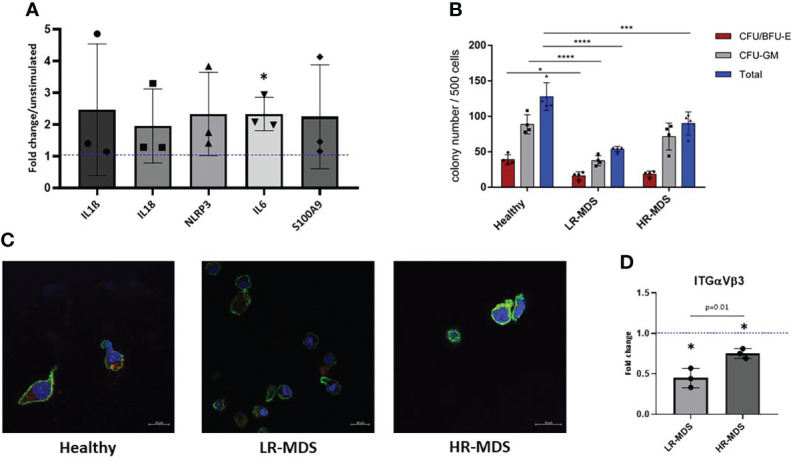
HSPC biology on MDS and healthy MSC matrices. **(A)** The expression of IL1b, IL18, NLRP3, IL6 and S100A9 in MNCs was evaluated after 24 hours of stimulation with low molecular HA. Relative target quantity was determined using the comparative CT (ΔΔCT) method. Amplicons were normalized to endogenous U6 expression and the untreated control was set to 1 (dotted line). Cumulative data from three different experiments are shown as mean ± SD. Significance was assessed by one-way ANOVA with Tukey’s multiple comparisons test, *p < 0.05. **(B)** A CFU-GEMM assay was performed for 14 days in methylcellulose medium, and the colonies were classified by using the StemVision system. The bars represent the mean ± SEM from four different donors in each group (n=3-4), *p < 0.05, ***p < 0.001, ****p < 0.0001 by two-way ANOVA with Tukey’s multiple comparisons test. **(C)** Representative confocal microscopy images of phalloidin (green) and ITGαVβ3 (red) with nuclear DAPI staining of HSPCs which were cultured on ECM derived from either healthy donors, LR- or HR-MDS MSCs. **(D)** Semi-quantitative analysis of relative fluorescence intensity of ITGαVβ3 determined by ImageJ analysis. Bars represent mean ± SD of fold changes compared to the healthy MSC ECM sample shown as a dotted line at an ordinate value of 1. Significant differences between MDS and healthy control as determined by one sample t-test are indicated by an asterisk above the bar: * = p< 0.05 and differences between the two MDS groups as determined by unpaired t-test are shown by the p value above the respective bars.

Both ECM stiffness and composition can impact the cellular behaviour. LMW-HA is known to play a role in inflammation (18) which is characteristic of LR-MDS BM. To test this hypothesis, BM MNCs from healthy donors were stimulated with LMW- HA (10 kDa) for 24 hours and the expression of various inflammatory genes was analyzed. We observed a significant increase in IL6 expression and a trend towards increased expression of IL1ß, IL18, NLRP3, PYCARD, and S100A9 upon stimulation with HA (**Figure 5A**).

### MDS ECM modulates HSPC biology

To further decipher the effects of disease-associated changes in ECM composition on CD34^+^ HSPCs, colony-forming unit (CFU) assays were performed after a seven day culture on healthy, LR- or HR-MDS MSC ECM. Interestingly, the number of colonies both for BFU/CFU-E and CFU-GM was significantly decreased in the group of HSPCs cultured on LR-MDS ECM (**Figure 5B**). Similar, but less pronounced effects were observed following culture on HR-MDS ECM (**Figure 5B**).

In addition to modulating the clonogenic capacity of HSPCs, the MSC-derived ECM also influenced their morphology. F-actin staining demonstrated a polarized morphology in HSPCs cultured on healthy ECM whereas cells on both LR- and HR-MDS matrix displayed a round, non-polarized shape (**Figure 5C**). This was accompanied by decreased integrin expression in cells cultured on MDS MSC-derived ECM (**Figures 5C, D**).

## Discussion

It has been recognized for some time that the BMME plays a role in MDS initiation and progression and MSCs as a major cellular component have been increasingly studied in this context (26–29). However, little is known about the role of MSC-derived ECM for the disease pathology.

By using a previously established *in vitro* approach to generate decellularized ECM scaffolds derived from MSCs (23), in this study we analyzed structural and functional characteristics of LR- and HR-MDS ECM in comparison to hematologically healthy control samples. We demonstrated alterations of MDS ECM in height and stiffness as well as in GAG composition which correlate to impaired interaction with normal CD34^+^ HSPCs.

The bone marrow ECM undergoes constant remodeling, the balance of which is essential for homeostasis. The stiffness of the ECM is heterogeneous throughout the BM (30), likely due to heterogeneous distribution of ECM components. We observed that MDS MSC-derived matrices were thicker and with more compact bundles than were matrices from healthy donors. However, MDS samples were softer than the controls. Both, height and stiffness modification were more pronounced in HR- than in LR-MDS-derived samples (**Figure 1**) suggesting a consistent remodeling during disease progression.

Many cell types respond to the stiffness of substrates and neighboring cells *in vitro* and most cell types increase adherent area on stiffer substrates that are coated with ligands for integrins or cadherins (31). *In vivo* cells engage their ECM by multiple mechanosensitive adhesion complexes and other surface receptors that potentially modify the mechanical signals transduced at the cell/ECM interface (31). In line with this, CD34^+^ HSPCs cultured on the different matrices showed less interaction with the softer MDS-derived ECM. Moreover, the expression of integrin αvβ3 was lower in cells with a round phenotype (**Figure 5**), possible as a result of diminished access of ligands.

Alongside the biochemical cues, stromal and hematopoietic function is governed by biophysical cues (8). These initiate outside-in signalling through integrin activation and focal adhesion complexes which may then influence lineage specification and morphological changes (8, 32, 33). In our study, the physical changes observed in the MDS MSC- derived ECM were associated with morphological modifications in hematopoietic cells cultured on the ECM. The dense and thick ECM deposited by MDS MSCs may hinder access to active binding sites and thus lead to a more rounded morphology of cells cultured on MDS ECM substrates. We suggest that these changes may lead to altered activation of integrins and other cell surface receptors regulating proliferation and differentiation pathways.

Our study also demonstrated changes in the biochemical composition of the ECM. Changes in ECM related-glycoprotein and collagen gene expression in MDS MSC have previously been reported (27, 34). However, since ECM synthesis is greatly dependent on post-translational modifications and non-template driven multistep enzymatic processes, so that biochemical analysis of GAG can reveal differences not necessarily evident from changes in gene expression (35). Therefore, analysis of proteoglycans can provide a more reliable indication of their involvement in malignant transformation. We show changes in ECM proteins, and especially GAGs as key part of proteoglycans known to control cell-cell and cell-ECM signalling, in MDS ECM. Decreased levels of *N*-acetyl-galactosamine GAGs, i.e. CS, were observed in HR-MDS, while LR-MDS MSCs showed the highest deposition (**Figure 2**). The L-type lectin PNA recognizes terminal β-galactose linked to *N*-acetyl-galactosamine (GalNAc) (36), while WGA binds to *N*-acetyl-glucosamine (GlcNAc) and sialic acid residues (37). PNA was shown to colocalize with CS proteoglycans (38). However, these carbohydrate motifs detected by PNA and WGA are not limited to carbohydrate structures present in GAG chains of proteoglycans, but are widely distributed among glycoproteins bearing *N*- or O-glycans (39). Thus, the detected increase in PNA and WGA staining in LR-MDS ECM may further point to alterations of glycoconjugates containing β-galactose, GlcNAc and sialic acid. This could attribute to the disease stage-specific changes in the MDS bone marrow. Katagiri et al. observed an increased BM Lin-/Sca1+/cKit+ cell fraction and HSPCs with a delayed short-term reconstitution in the T1KO mouse model which has reduced levels of CS (40). The reduced deposition of CS in HR-MDS-derived ECM may contribute to the increased cellularity observed in HR-MDS bone marrow. However, the CS-56 antibody staining has some limitations. The detected alterations can result from either i) a decreased production of CS chains and/or CS proteoglycans and/or ii) differences in the sulfation pattern within the CS chains recognized by the CS-56 antibody.

Our study also demonstrated changes in the HA content of MDS ECM, with the highest levels being present in samples from LR-MDS patients (**Figure 3**). A previous study of BM serum has reported increased HA levels in HR-MDS samples (41). Since our study focuses on HA bound to matrix deposited by MSCs, we cannot rule out that other cells contribute to the soluble HA found in BM serum. However, the high HA levels that we find in the matrix would be consistent with the increased and dense ECM deposition by MDS MSCs. It is also possible that an increase in hydration due to HA contributes to the decreased rigidity of MDS ECM, although this may also be due to changes in the crosslinking of the ECM proteins. It is worth noting that HA can augment or override mechanical signaling by some classes of integrins to produce a cellular phenotype that is otherwise observed only on very rigid substrates (31).

HA produced by the MSCs is known to support hematopoietic activity *in vivo* (42). However, the effects of HA are strongly dependent on molecular weight, with high molecular weight HA having anti-inflammatory activity and low molecular weight possessing pro-inflammatory properties (18, 43, 44). The presence of LMW-HA has been observed during malignant transformation in various solid tumours (45, 46). Consistent with these previous observations, we find that LMW-HA is present at high levels in LR-MDS ECM and can induce IL-6 expression in hematopoietic cells. Since IL-6 levels are known to be increased in LR-MDS marrow, our findings suggest the potential contribution of LMW HA to the inflammatory microenvironment in LRMDS.

## Conclusion

In summary, we provide the first evidence for structural alterations of the MSC-derived ECM in both LR- and HR-MDS and imply a decisive role for GAGs in remodelling of the matrix and changing hematopoietic support properties to support malignant transformation.

## Data availability statement

The original contributions presented in the study are included in the article/**Supplementary Material**. Further inquiries can be directed to the corresponding author.

## Ethics statement

The studies involving human participants were reviewed and approved by Ethikkommission TU Dresden. The patients/participants provided their written informed consent to participate in this study.

## Author contributions

AKB, LW, JR, SR, VM, MC, and UP contributed to conception and design of the study. AB, LW, SR, VM, and JF performed experiments and data analysis. AB and MW wrote the manuscript. LW, JR, SR, VM, JF, MB, MC, KG, and UP edited the manuscript. All authors contributed to manuscript revision, read, and approved the submitted version.

## Acknowledgments

The authors would like to thank Mandy Richter, Robert Kuhnert, Kristin Möbus, Katrin Müller, Dennis Ossman, as well as the Jose Carreras Leukämie Stiftung (DJCLS 03 R /2018 to UP, MW and CW) and the Core Facility Cellular Imaging of TU Dresden. We thank Dunya Günay for excellent technical assistance during the electrophoresis part as well as Nelly Rein for POMA slide preparation.

## Conflict of interest

The authors declare that the research was conducted in the absence of any commercial or financial relationships that could be construed as a potential conflict of interest.

## Publisher’s note

All claims expressed in this article are solely those of the authors and do not necessarily represent those of their affiliated organizations, or those of the publisher, the editors and the reviewers. Any product that may be evaluated in this article, or claim that may be made by its manufacturer, is not guaranteed or endorsed by the publisher.
