# Supplementary figures and images for: Bone marrow mesenchymal stromal cell-derived extracellular matrix displays altered glycosaminoglycan structure and impaired functionality in Myelodysplastic Syndromes
Source: Front Oncol. 2022 Sep 8;12:961473. doi: 10.3389/fonc.2022.961473 (PMC9492883; doi:10.3389/fonc.2022.961473)

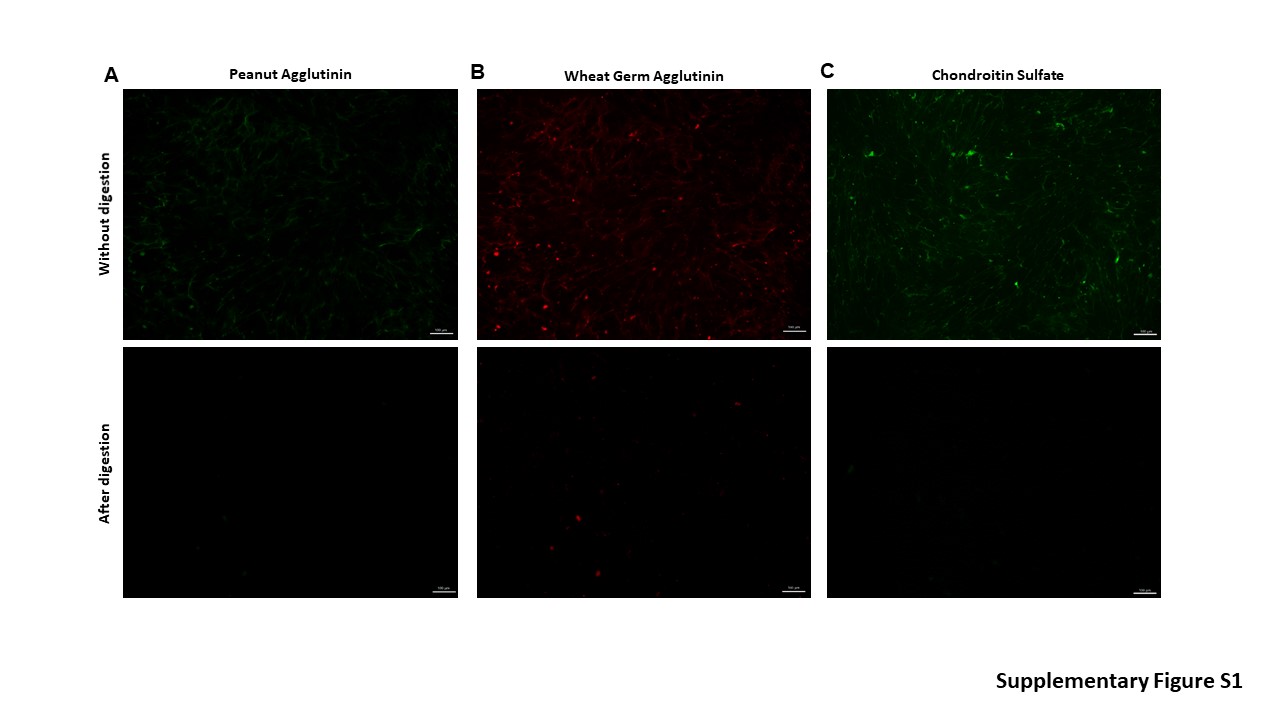

Supplement: Supplementary file 1 [file Image_1.jpeg]
