# Supplementary material for: Bone marrow mesenchymal stromal cell-derived extracellular matrix displays altered glycosaminoglycan structure and impaired functionality in Myelodysplastic Syndromes
Source: Front Oncol. 2022 Sep 8;12:961473. doi: 10.3389/fonc.2022.961473 (PMC9492883; doi:10.3389/fonc.2022.961473)
Supplement: Supplementary file 2 [file DataSheet_1.docx]

**Supplementary Material**

**supplementary figure captions**

**Supplementary Figure S1. Analysis of *N*-acetyl-glucosamine and *N*-acetyl-galactosamine GAGs as well as of CS in ECM after pretreatment chondroitinases ABC and heparinise-1, respectively.**

Representative images of HR-MDS MSC ECM from the lectin staining using Wheat germ agglutinin (A) which binds to *N*-acetyl-glucosamine GAGs (HA, heparan sulfate and keratan sulfate) and sialic acid and peanut agglutinin (B) which binds to *N*-acetyl-galactosamine and terminal ß-galactose containing GAGs (CS, dermatan sulfate and keratan sulfate) without or with enzymatic pretreatment with chondroitinases ABC and heparinise-1. (C) Representative images from chondroitin sulfate immunostaining of HR-MDS MSC ECM without or with enzymatic pretreatment with chondroitinases ABC.

**supplementary tables**

**Supplementary Table S1. MDS patient information**

| **No.** | **Sex** | **Age** | **IPSS** | **IPSSR** | **Sample type** |
| --- | --- | --- | --- | --- | --- |
| **1** | M | 59 | High | High | MSC |
| **2** | M | 62 | Int-2 | High | MSC |
| **3** | M | 62 | Very high | High | MSC |
| **4** | M | 61 | Very high | High | MSC |
| **5** | M | 73 | Int -2 | High | MSC |
| **6** | M | 69 | Int | Int-1 | MSC |
| **7** | F | 66 | Int | Int -1 | MSC |
| **8** | M | 63 | Low | Low | MSC/MNC |
| **9** | M | 67 | Int | Int -1 | MSC/MNC |
| **10** | M | 69 | Int -1 | Low | MSC/MNC |

**Supplementary Table S2. Primer sequences**

| **Target** | **Foward primer (5´- 3´)** | **Reverse Primer(5´- 3´)** |
| --- | --- | --- |
| **hHAS1** | CGGAGATTCGGTGGACTACG | CCCAGGAGTCCAGAGGGTTA |
| **hHAS2** | GTCGAGTTTACTTCCCGCCA | ATCACACCACCCAGGAGGAT |
| **hHAS3** | GGTCATGTACACGGCCTTCA | CCAGGACTCGAAGCATCTCG |
| **hCSGALNACT1** | CATGGCCAACACGCTTATCA | CTGAAGTTGGCAGCTTTGGAAG |
| **hIL6** | TGGCAGAAAACAACCTGAACC | CCAGTGATGATTTTCACCAGGC |
| **hIL1ß** | TGATGGCTTATTACAGTGGCA | GGTGGTCGGAGATTCGTAGC |
| **hIL18** | TGCAGTCTACACAGCTTCGG | ACTGGTTCAGCAGCCATCTT |
| **hNLRP3** | CAAGCAAGATGCGGAAGCTC | GTCCTCCACCAGGTAGGACT |
| **hS100A9** | CGGCTTTGACAGAGTGCAAG | GCCCCAGCTTCACAGAGTAT |
| **hDSE** | GGAAACAACTTGGGTGCCTTG | GACCTCATCCCAAGGAGCATC |
| **hEXT-1** | TGCCTGTCGTCGTCATTGAA | AGGCGAAATCCACCTCTGTT |
| **hCHSY1** | GCACGACCACTACTTGGACA | TCGCTGCTGTTCAAACTCCT |
| **hCHST11** | ATGCGGAGGAATCCCTTT | GCAGGACAGCAGTGTTTGAG |
| **hHYAL-1** | ACACGACAAACCACTTTCTGC | GCCCCAGTGTAGTGTCCATA |
